# MFAP5 as a promising biomarker for connective tissue disease-associated interstitial lung disease

**DOI:** 10.3389/fimmu.2025.1642408

**Published:** 2025-10-02

**Authors:** Yufang Dai, Jiaqian Zhang, Xiufeng Bai, Shasha Wu, Yanqiong Chen, Dachao Mou, Yun Wang, Yunlong Zhu, Yi Liu

**Affiliations:** ^1^ Department of Rheumatology and Immunology, West China Hospital, Sichuan University, Chengdu, Sichuan, China; ^2^ Minda Hospital of Hubei Minzu University, Hubei Provincial Key Laboratory of Occurrence and Intervention of Rheumatic Disease, Enshi, Hube, China; ^3^ Laboratory of Human Disease and Immunotherapies, West China Hospital, Sichuan University, Chengdu, Sichuan, China

**Keywords:** MFAP5, CTD, ILD, potential, biomarker

## Abstract

Interstitial lung disease (ILD), a common and severe complication of connective tissue disease (CTD), can cause progressive lung function decline and even death. However, current biomarkers for diagnosing and predicting CTD-ILD are unsatisfactory. Here, we identify a new diagnostic and prognostic biomarker for CTD-ILD. We used comparative transcriptomic sequencing and bioinformatics analysis of the Gene Expression Omnibus (GEO) database to identify upregulated genes in lung fibroblasts of systemic sclerosis-associated ILD. Peripheral blood, bronchoalveolar lavage fluid (BALF), and lung tissue samples from healthy donors, CTD patients, and CTD-ILD patients were collected. Immunohistochemistry, immunofluorescence, and ELISA were used to validate the expression levels of candidate biomarkers. Microfibril-Associated Protein 5 (MFAP5) is upregulated in the lung tissue of ILD patients. Meanwhile, serum and BALF MFAP5 levels in CTD-ILD patients are significantly elevated compared to those in CTD patients without ILD and healthy controls, showing positive correlations with the extent of ILD. involvement and multiple inflammatory markers, along with a negative correlation with anti-inflammatory immunoglobulin IgG. MFAP5 has 89.53% specificity in differentiating CTD-ILD from CTD without ILD. Furthermore, in the bleomycin (BLM)-induced mouse model, MFAP5 mRNA and protein expression were increased. These findings suggest that MFAP5 levels are elevated in CTD-ILD patients and may serve as a biomarker for diagnosing and predicting CTD-ILD.

## 1 Introduction

Connective tissue diseases (CTDs) comprise a complex group of autoimmune disorders, including systemic lupus erythematosus (SLE), systemic sclerosis (SSC), rheumatoid arthritis (RA), mixed connective tissue disease (MCTD), primary Sjögren’ s syndrome (PSS), and idiopathic inflammatory myopathies (IIM). While these conditions exhibit distinct pathological mechanisms and clinical manifestations, they share a tendency to induce severe pulmonary complications, particularly interstitial lung disease (ILD). ILD encompasses a broad spectrum of fibrotic and inflammatory lung conditions characterized by diverse pathological processes. Over the past decade, the global incidence of ILD has significantly increased by 51% (1). Among ILD patients, connective tissue disease-associated interstitial lung disease (CTD-ILD) makes up a substantial proportion, serving as a common and life-threatening pulmonary complication of CTDs.

In different subtypes of CTD-ILD, the reported prevalence of systemic sclerosis-associated ILD (SSc-ILD) varies significantly across studies, ranging from 26.1% to 88.1%. It is one of the leading causes of death in patients with systemic sclerosis (2). In contrast, the prevalence of rheumatoid arthritis (RA)-associated ILD is relatively low, at only 0.6% (3). Approximately 20% of patients with PSS develop ILD. Among patients with idiopathic inflammatory myopathy, 36%-45% have ILD (4), and this proportion can significantly increase to 80% in those with anti-synthetase antibodies (5, 6). Additionally, there are striking geographical disparities in the prevalence of myositis-associated ILD, with 23% in the United States and as high as 50% in Asia (7), 1%-2% of patients with SLE develop ILD as a complication (8). Among those diagnosed with fibrotic ILD (fILD), when they died from any cause, the fILD itself was the underlying cause of death in up to 45% of these patients (9).

ILD is classified into two pathological patterns: ILD (no fibrosis) and fILD. ILD and fILD are not entirely distinct diseases but exist on a disease spectrum with interrelated pathophysiology and potential for clinical progression (10, 11). The former represents an “early/mild stage,” while the latter signifies an “advanced/severe stage (12),” with the two closely linked through “inflammation-driven fibrotic transformation (11, 13).” Approximately 30-40% of ILD cases progress to fILD, leading to irreversible functional decline and reduced quality of life (14). fILD is the most common and severe form, characterized by a poor prognosis, with primary symptoms including dyspnea, exercise intolerance, and cough. The median survival period ranges from 3 to 7 years (15), and the 5-year survival rate is below 50% (16, 17).

ILD is marked by dyspnea, a gradual decline in lung function, and a poor prognosis (18). Since early diagnosis and monitoring of ILD are crucial, current studies have identified numerous novel candidate biomarkers for ILD, including matrix metalloproteinase 7 (19, 20), surfactant proteins A and D (21–23), Krebs von den Lungen-6 (24, 25), chemokine ligand 18 (26, 27), chitinase-3-like protein 1 (28), and Mucin 5B (29). While these biomarkers enhance diagnostic sensitivity and prognostic accuracy for ILD, there is still insufficient evidence to support the translation of these biomarkers into clinical practice. Current diagnostic approaches for ILD predominantly rely on pulmonary high-resolution computed tomography (HRCT). However, the sensitivity of imaging for early-stage ILD detection remains suboptimal. Furthermore, once fibrotic lesions are established, CT imaging often lacks the discriminative capacity to reliably assess short-term disease progression or stabilization. In contrast, serum biomarkers demonstrate detectable elevation prior to the manifestation of radiological abnormalities, offering potential for detection and longitudinal monitoring of disease activity and therapeutic response.

The activation of fibroblasts and their trans-differentiation into myofibroblasts, leading to aberrant extracellular matrix deposition and structural remodeling, represent key pathogenic mechanisms in ILD (30). Pathophysiologically, SSc-ILD shares mechanistic similarities with idiopathic pulmonary fibrosis (31) and post-COVID-19 fibrosis (32), characterized by chronic alveolar-capillary inflammation and progressive fibrosis. To further investigate the pathogenesis of CTD-ILD and identify key molecules, transcriptome sequencing and bioinformatics analysis were performed to compare differentially expressed molecules between fibroblasts from SSc-ILD patients and healthy human lung fibroblasts. Fibrotic fibroblasts demonstrated significantly elevated expression of MFAP5 compared to healthy controls. Although the expression of MFAP5 is elevated in this disease, its specific mechanism of action in ILD remains unclear. Therefore, in-depth research on the expression level of MFAP5 in ILD and its clinical relevance will help clarify its potential role in disease progression and provide new directions for the disease’s condition assessment and treatment strategies.

## 2 Materials and methods

### 2.1 Bioinformatics analysis

Key terms “systemic sclerosis AND pulmonary fibrosis” and species “*Homo sapiens*” were used to search the NCBI GEO database ( *https://www.ncbi.nlm.nih.gov/geo/* ), yielding a dataset (GSE215841) comparing whole transcriptome expression profiles between normal lung fibroblasts and systemic sclerosis patient derived fibroblasts. Detailed dataset information is provided in Data Sheet 3. Differential expression analysis was performed using GEO2R ( *https://www.ncbi.nlm.nih.gov/geo/geo2r/?acc=GSE21584* ) with criteria of |logFC| > 1 and adjusted *P*<0.05, followed by visualization of results via a volcano plot. Pathway enrichment analysis of differentially expressed genes was conducted using Metascape ( *http://metascape.org* ). Genes with significant upregulation in disease states were selected for further evaluation. PubMed ( *https://pubmed.ncbi.nlm.nih.gov/* ) was queried to assess the feasibility and novelty of candidate genes (Data Sheet 4).

### 2.2 Clinical patient

A total of 97 CTD without ILD patients and 169 CTD-ILD patients were enrolled between December 31, 2023, and December 31, 2024, at the Minda Hospital of Hubei Minzu University, alongside 113 healthy controls (HC) recruited via a physical examination center. CTD diagnoses followed the 2013 American College of Rheumatology (ACR)/European League Against Rheumatism (EULAR) criteria (33–37) while CTD-ILD patients met radiological criteria for ILD on high resolution computed tomography, including diffuse ground glass opacities, reticular opacities, traction bronchiectasis, or honeycombing (38). The extent of ILD was assessed using a validated semi-quantitative HRCT scoring system, which categorized the involvement into four grades: Grade 1 (0-25%), Grade 2 (26-50%), Grade 3 (51-75%), and Grade 4 (76-100%) (39). To minimize inter-observer variability, HRCT scoring was independently performed by two radiologists and two senior respiratory physicians, with final scores calculated as the mean of the four assessments.

Peripheral serum samples were acquired from healthy volunteers, CTD-ILD patients, and CTD patients without ILD. Comprehensive clinical data were systematically recorded for all participants. Exclusion criteria for the disease group: (1) other pulmonary diseases (e.g., tumors, bronchiectasis, COPD, tuberculosis); (2) non-CTD-ILD (e.g., pneumoconiosis, radiation pneumonitis); (3) severe organ dysfunction (cardiac/renal failure); (4) prior malignancy; (5) pregnancy/lactation; (6) infectious diseases (hepatitis, syphilis, HIV); (7) neuropsychiatric disorders or refusal to consent; (8) smoking history. Healthy controls, matched for age and sex, were recruited from a physical examination center, with inclusion criteria including age between 18 and 80 years, absence of underlying diseases, no smoking history, and normal blood counts, liver function, and kidney function. Exclusion criteria included age <18 or >80 years, pregnancy, psychiatric disorders, or recent medication use. The inclusion criteria for CTD-ILD complicated by infection were a confirmed diagnosis of CTD-ILD, fever (temperature >37.5°C, non-drug-induced), new-onset or worsening cough with purulent sputum, new pulmonary rales or wheezing, white blood cell count (WBC) >10×10^9^/L or <4×10^9^/L with neutrophilia, C-reactive protein (CRP) >10 mg/L, and procalcitonin (PCT) >0.5 ng/mL. BALF was collected from CTD-ILD patients with pulmonary infections and from individuals with isolated pulmonary infections. Exclusion criteria included acute exacerbation of ILD without evidence of infection, pulmonary embolism, and drug-induced lung injury. In this study, patients with pulmonary infection (rather than ILD) were selected as the BALF control group. The primary purpose of this selection is to provide a reference baseline that can match the inflammatory background of ILD complicated with infection.

Human lung tissue samples were sourced from paraffin blocks of patients with CTD-ILD who underwent lung tissue biopsy for other diseases and whose samples were preserved in the hospital. Healthy lung tissue was obtained from paraffin blocks of lung nodules that were surgically resected, with the final diagnosis being benign nodules, and the tissue was taken from the area adjacent to the nodules. The study protocol was approved by the Ethics Committee of Minda Hospital, Hubei Minzu University (Approval No. 2024004), and written informed consent was obtained from all participants.

### 2.3 Animal model establishment

Eight-week-old male C57BL/6 mice (body weight 20-22g) were purchased from Chengdu Kemeixin Biotechnology (Chengdu, China) and housed under specific pathogen-free conditions. After 1 week of acclimation with ad libitum access to food and water, mice were randomized into two groups: bleomycin (BLM)-treated (4 mg/kg intratracheal instillation) and saline control (isovolumetric saline), with 6-8 animals per group. Mice were anesthetized with isoflurane. After cervical incision, BLM (4 mg/kg dissolved in saline) or saline alone was instilled into the trachea. Wounds were sutured, and animals were placed on a heating pad until recovery. At specified time points (day 0, 7, 14, 21, and 28 post-instillation), mice were euthanized under deep isoflurane anesthesia. Peripheral blood and lung tissues were collected for analysis. All animal experiments were performed in accordance with guidelines from the Institutional Animal Care and Use Committee, with protocols approved by West China Hospital, Sichuan University (Approval No. 20230427002).

### 2.4 Immunohistochemistry staining

Lung tissue sections were dewaxed in xylene, hydrated through graded ethanol washes, and rinsed with PBS buffer. Antigen retrieval was performed via microwave boiling followed by natural cooling to room temperature and PBS rinsing. Endogenous peroxidase activity was quenched with 3% hydrogen peroxide, and sections were again rinsed with PBS. Non-specific binding sites were blocked with goat serum for 20-30 minutes at room temperature. Sections were then incubated overnight with MFAP5 primary antibody (1:200 dilution, Abcam: ab232846). After washing, biotinylated secondary antibody (1:200) was applied, followed by streptavidin-peroxidase complex incubation for 30 minutes at room temperature. Diaminobenzidine (DAB) chromogen was used for visualization, with reactions terminated by distilled water rinsing. Sections were counterstained with hematoxylin, dehydrated, and mounted with neutral resin. ILD severity was individually graded using Ashcroft’s semi-quantitative system (40).

### 2.5 Indirect immunofluorescent staining

Lung tissue sections were sequentially dewaxed in xylene, hydrated through graded ethanol washes, and rinsed with PBS buffer. Antigen retrieval was performed via microwave boiling followed by natural cooling to room temperature and PBS rinsing. Sections were blocked with immunofluorescence blocking solution (Beyotime, P0102) for 1 hour at room temperature. Primary antibodies (MFAP5 1:200 dilution, Abcam: ab232846, ab203828) were applied and incubated overnight at 4°C. After PBS rinsing, sections were incubated with Alexa Fluor™ 488-conjugated goat anti-rabbit secondary antibody (1:200 dilution, Invitrogen) for 1 hour at room temperature. Nuclei were counterstained with DAPI for 5 minutes, and sections were mounted with antifade mounting medium and coverslips, sealed with neutral resin.

### 2.6 Masson’s trichrome stain

Lung tissue sections were sequentially dewaxed in xylene, hydrated through graded ethanol, rinsed with PBS buffer, and stained with Masson’s Trichrome Stain kit. Nuclear staining was performed with Weigert’s Hematoxylin Staining Solution for 5-10 minutes. Sections were differentiated in 1% hydrochloric acid in ethanol for 5-15 seconds, followed by bluing in 1% lithium carbonate solution for 1 minute. Masson’s ponceau-acid fuchsin solution was applied for 5-10 minutes, and slides were rinsed in 2% glacial acetic acid for 1 minute. Differentiation was carried out in 1% phosphomolybdic acid aqueous solution for 3-5 minutes, followed by another 1-minute rinse in 2% glacial acetic acid. Counterstaining with 1% light green aqueous solution was performed for 1-2 minutes. After a final 0.2% glacial acetic acid rinse, sections were dehydrated in ethanol, cleared in xylene, and mounted with neutral resin.

### 2.7 RNA extraction and quantitative polymerase chain reaction

Total mRNA was extracted from cells and mouse lung tissues using Trizol reagent (Vazyme, R401-01). Complementary DNA (cDNA) was synthesized via reverse transcription with a Takara kit, followed by cDNA amplification using SYBR Green Master Mix (Takara). Primers used in qPCR are listed in Data Sheet 1 and were synthesized by Tsingke Biotech. Relative target gene expression was normalized to 18S ribosomal RNA levels and calculated using the 2(-△△CT) method.

### 2.8 Western blot

Mouse lung tissues were lysed with RIPA lysis buffer containing protease/phosphatase inhibitors (Beyotime) for 30 minutes, followed by centrifugation to collect supernatants. Protein concentrations were determined using a BCA protein assay kit (Beyotime). Lysates were boiled in loading buffer for 10 minutes, separated via SDS-PAGE (20-30μg protein per lane), and transferred to PVDF membranes (Millipore). Membranes were blocked with 5% non-fat milk at room temperature for 1 hour, incubated with primary antibodies overnight at 4°C, washed three times with TBST, and probed with species-specific HRP-conjugated secondary antibodies for 1 hour at room temperature. Protein bands were visualized using a Bio-Rad imaging system and quantified with ImageJ 1.8.0 software. Antibodies used included: anti-MFAP5 (1:1000 dilution, Abcam: ab232846) and β-Tubulin (1:5000 dilution, HUABIO: ET1602-4) (Data Sheet 2).

### 2.9 Enzyme-linked immunosorbent assay

Human MFAP5 levels were measured using a commercial ELISA kit (E20250102-RX100335H, RUIXIN Biotech, Fujian, China). Test samples were added to the solid-phase carrier pre-coated with anti-MFAP5 antibodies, allowing target molecules to bind. After washing, enzyme-conjugated secondary antibodies were added to specifically recognize bound MFAP5. Unbound conjugates were removed via washing, followed by the addition of chromogenic substrate solution. Enzymatic reactions were visualized by color development, and optical density values were measured at 450 nm using a microplate reader.

### 2.10 Statistical analysis

Statistical analyses were performed using GraphPad Prism 9.5 (GraphPad Software, Inc., CA, USA) and SPSS 26.0 (IBM Corp., NY, USA). Normally distributed continuous variables were compared using independent samples t-tests (two groups) or one-way analysis of variance (ANOVA) with *post-hoc* testing (multiple groups). Non-normally distributed data were presented as median and interquartile range (IQR), analyzed via Mann-Whitney U tests. Correlations were evaluated using Spearman rank correlation coefficients. Study participants were divided into positive and negative groups, which included three comparison pairs: healthy control group vs. CTD-non-ILD group, healthy control group vs. CTD-ILD group, and CTD-ILD group vs. CTD-non-ILD group. The Area Under the ROC Curve (AUC) was used to quantify the overall discriminative efficacy, with the following interpretive criteria: an AUC of 0.5 indicates no discriminative value, an AUC between 0.7 and 0.8 (inclusive of 0.7, exclusive of 0.8) indicates good discriminative efficacy, and an AUC between 0.8 and 0.9 (inclusive of 0.8, exclusive of 0.9) indicates excellent discriminative efficacy. Based on the “Thresholds” table output by SPSS, the optimal diagnostic threshold was determined using the method of maximizing the Youden’s Index (Youden’s Index= Sensitivity+Specificity-1). Meanwhile, the sensitivity and specificity corresponding to this optimal threshold were extracted. Statistical significance was defined as two-tailed *P*<0.05, denoted as: *P<0.05, **P<0.01, ***P <0.001, with “ns” indicating non-significance.

## 3 Results

### 3.1 MFAP5 expression was increased in serum and alveolar lavage fluid of CTD-ILD

**Figure 1:**
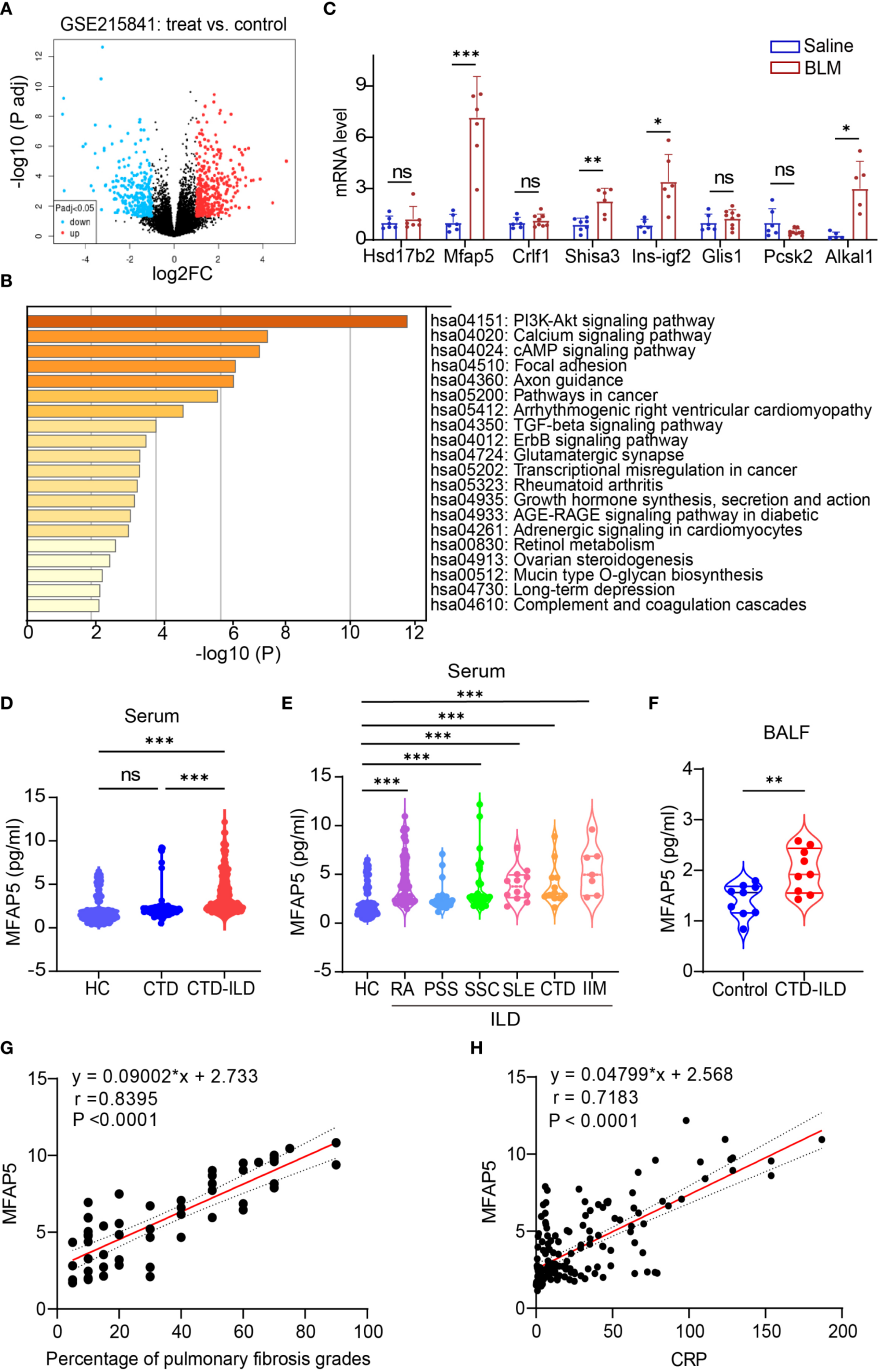
Identification and validation of differentially expressed genes. **(A)** Volcano plot showing gene expression levels in lung tissues of patients vs. healthy controls: colored dots represent DEGs, with red (upregulated), blue (downregulated), and gray (no change). **(B)** KEGG pathway enrichment analysis of DEGs. **(C)** qPCR validation of candidate genes in BLM-induced ILD vs. control mouse lungs (n=5-9 independent experiments). **(D)** ELISA quantification of serum MFAP5 levels in HC, CTD, and CTD-ILD patients. **(E)** MFAP5 expression differences across CTD-ILD subtypes and HC. **(F)** ELISA detection of MFAP5 in BALF of pulmonary infection patients with or without CTD-ILD. **(G)** Correlation analysis between MFAP5 and CTD-ILD pulmonary involvement severity. **(H)** Correlation between MFAP5 and serum CRP in CTD-ILD patients. Data are presented as mean ± standard deviation. ns, not significant; *P < 0.05, **P < 0.01, ***P < 0.001.

We first analyzed mRNA expression profiles between SSc-PF samples and normal controls from public datasets. The GSE215841 dataset included 23 samples: 12 lung fibroblasts from SSc-PF patients and 11 from healthy donors. GEO2R analysis of transcriptome sequencing data identified 1,6248 genes, with 364 upregulated and 336 downregulated (**Figure 1A**). Kyoto Encyclopedia of Genes and Genomes (KEGG) pathway enrichment analysis of these DEGs highlighted significant enrichment in PI3K-AKT and TGF-β signaling pathways (**Figure 1B**). The PI3K-AKT pathway regulates core cellular processes, including growth, proliferation, and survival (41), The TGF-β/SMAD signaling pathway plays a pivotal role in pulmonary fibrosis pathogenesis (42), with excessive TGF-β production driving pathological scarring and extracellular matrix deposition (43). Crosstalk between TGF-β and PI3K/AKT signaling further promotes fibrogenesis (44), providing a mechanistic foundation for downstream pathway analysis. Based on literature review, Gene Cards analysis, and novelty assessment, 10 candidate genes were shortlisted (Data Sheet 4). These were validated in a BLM-induced ILD mouse model via qPCR. Among them, MFAP5 showed the most significant differential expression, leading to its selection as the target molecule (**Figure 1C**).

The study included 97 CTD patients, 169 CTD-ILD patients (sub-grouped as 82 RA-ILD, 20 PSS-ILD, 35 SSc-ILD, 12 SLE-ILD, 12 undifferentiated CTD-ILD, and 7 idiopathic inflammatory myopathy-ILD (IIM-ILD), Additionally, BALF samples were collected from 18 patients with pulmonary infection, 9 of whom had concurrent CTD-ILD. Demographics were balanced across groups: median age (interquartile range) was 62 (56,69) years for CTD, 56.5 (51,63) years for CTD-ILD, and 53 (46,61) years for HC. Statistical analysis showed no significant differences in median age or sex distribution (female proportion, *P *> 0.05). between CTD and HC groups, confirming demographic comparability (**Table 1**). Serum MFAP5 levels were significantly higher in CTD-ILD patients [2.82 (2.27, 5.27) pg/mL] than in CTD patients without ILD [1.99 (1.69, 2.30) pg/mL] (P<0.001), which, in turn, were markedly elevated compared with healthy controls [1.52 (1.025, 2.05) pg/mL] (P<0.001) (**Figure 1D**). No significant difference in MFAP5 expression was observed across CTD-ILD subtypes (**Figure 1E**). CTD-ILD patients exhibited significantly higher levels of Age, MFAP5, WBC, ALB, LDH, HBDH, D-dimer, CRP, ESR, and PCT compared to CTD-non-ILD patients (*P *< 0.05). with significantly higher expression in BALF of CTD-ILD patients with pulmonary infection (**Figure 1F**). MFAP5 positively correlated with degree of ILD involvement (**Figure 1G**) and serum CRP (**Figure 1H**).

**Table 1:** Demographic and baseline characteristics of study participants.

Characteristics	CTD (n=97)	CTD-ILD (n=169)	z/t	*p value*
Female, no. (%)	76(78.35)	123(72.78)	0.86	0.389
Age, year	56.5(51,63)	62(56,69)	4.38***	0.000
Duration of CTD, month	4(1,10)	3(1,7)	1.47	0.141
MFAP5, pg/mL	1.99(1.69,2.3)	2.82(2.27,5.27)	8.54***	0.000
RBC,1×10^12^/L	4.1(3.69,4.38)	4.03(3.67,4.45)	0.01	0.995
Hb,g/L	119(107,129)	119(106.5,130.5)	0.21	0.835
WBC, 1×10^12^/L	4.83(3.66,6.65)	6.22(4.97,7.53)	4.69***	0.000
PLT, 1×10^9^/L	213(179.5,293.5)	236(180,328.5)	1.12	0.261
TBIL, umol/L	8.1(6.65,11.05)	8.5(6.2,11.3)	0.12	0.905
DBIL, umol/L	3(2.5,4.1)	3.2(2.4,4.1)	0.20	0.839
ALT, U/L	14(10.4,20.35)	13(9.55,19)	1.46	0.144
AST, U/L	18(15,24)	19(15,25)	1.08	0.278
TP, g/L	64.59 ± 7.3	63.05 ± 8.32	1.51	0.132
ALB, g/L	37.7(34.45,40.05)	35.95(33.2,39.18)	2.63**	0.009
GLB, g/L	27.19 ± 6	27.45 ± 6.36	0.32	0.750
Glu, mmol/L	4.96(4.52,5.37)	4.76(4.33,5.37)	1.49	0.136
UREA, mmol/L	5.5(4.52,6.73)	5.45(4.69,7.12)	0.14	0.892
CREA, μmol/L	64.3(53.08,73)	64.2(53.5,70.88)	0.53	0.594
eGFR, ml/min	93.22 ± 17.35	91.08 ± 17.55	0.96	0.339
TG, mmol/L	1.08(0.75,1.47)	1.16(0.85,1.8)	1.82	0.068
CHOL, mmol/L	4.13(3.48,5.02)	4.22(3.61,5.15)	0.81	0.420
ALP, U/L	80.5(63.25,94)	76(64,96)	0.25	0.802
GGT, U/L	17.5(12.25,29)	20(14,34)	2.09*	0.037
CK, U/L	60(44.25,84.25)	50(33.25,92)	1.49	0.137
LDH, U/L	175(159.25,201.5)	196.5(165.75,236.75)	3.27**	0.001
HBDH, U/L	132.86 ± 32.87	151.5 ± 56.3	3.39**	0.001
D-dimer, μg/ml	0.56(0.31,1.62)	1.32(0.69,2.21)	3.89***	0.000
CRP, mg/L	3.33(0.8,15.73)	12.78(3.85,39.68)	4.77***	0.000
RF, IU/L	47.08(11.33,126.24)	51.97(10.46,249.84)	0.76	0.446
IgG, g/L	11.24(8.72,15.84)	12.18(9.74,16.98)	1.18	0.238
IgA, g/L	2.02(1.49,3.42)	2.25(1.68,3.07)	0.42	0.676
IgM, g/L	1.04(0.65,1.42)	1.15(0.73,1.66)	1.58	0.113
C3, mg/dL	1.17(1.07,1.3)	1.19(1.09,1.31)	0.64	0.520
C4, mg/dL	0.25(0.2,0.28)	0.23(0.19,0.3)	0.09	0.930
C1q, mg/dL	19.1(15.9,24.5)	20.1(17.2,30.28)	1.63	0.103
ESR, mm/h	43(20.5,84)	69(37.75,100.25)	3.40**	0.001
IL6, pg/ml	7.4(5.65,30.05)	10.58(6.3,35.6)	1.38	0.169
PCT, ng/ml	0.25(0.21,0.37)	0.33(0.27,0.38)	2.12*	0.034

RBC, Red Blood Cell Count; Hb, Hemoglobin; WBC, White Blood Cell Count; PLT, Platelet Count; TBIL, Total Bilirubin; DBIL, Direct Bilirubin; ALT, Alanine Aminotransferase; AST, Aspartate Aminotransferase; TP, Total Protein; ALB, Albumin; GLB, Globulin; Glu, Glucose; UREA, Urea; CREA, Creatinine; eGFR, Estimated Glomerular Filtration Rate; TG, Triglyceride; CHOL, Total Cholesterol; ALP, Alkaline Phosphatase; GGT, Gamma-Glutamyl Transferase; CK, Creatine Kinase; LDH, Lactate Dehydrogenase; HBDH, α-Hydroxybutyrate Dehydrogenase; D-dimer, D-Dimer; CRP, C-Reactive Protein; RF, Rheumatoid Factor; IgG, Immunoglobulin G; IgA, Immunoglobulin A; IgM, Immunoglobulin M; C3, Complement C3; C4, Complement C4; C1q, Complement component 1q; ESR, Erythrocyte Sedimentation Rate; IL6, Interleukin-6; PCT, Procalcitonin.

*P < 0.05, **P < 0.01, ***P < 0.001.

### 3.2 Serum MFAP5 levels correlate with clinical parameters and diagnostic prediction in CTD-ILD patients

**Figure 2:**
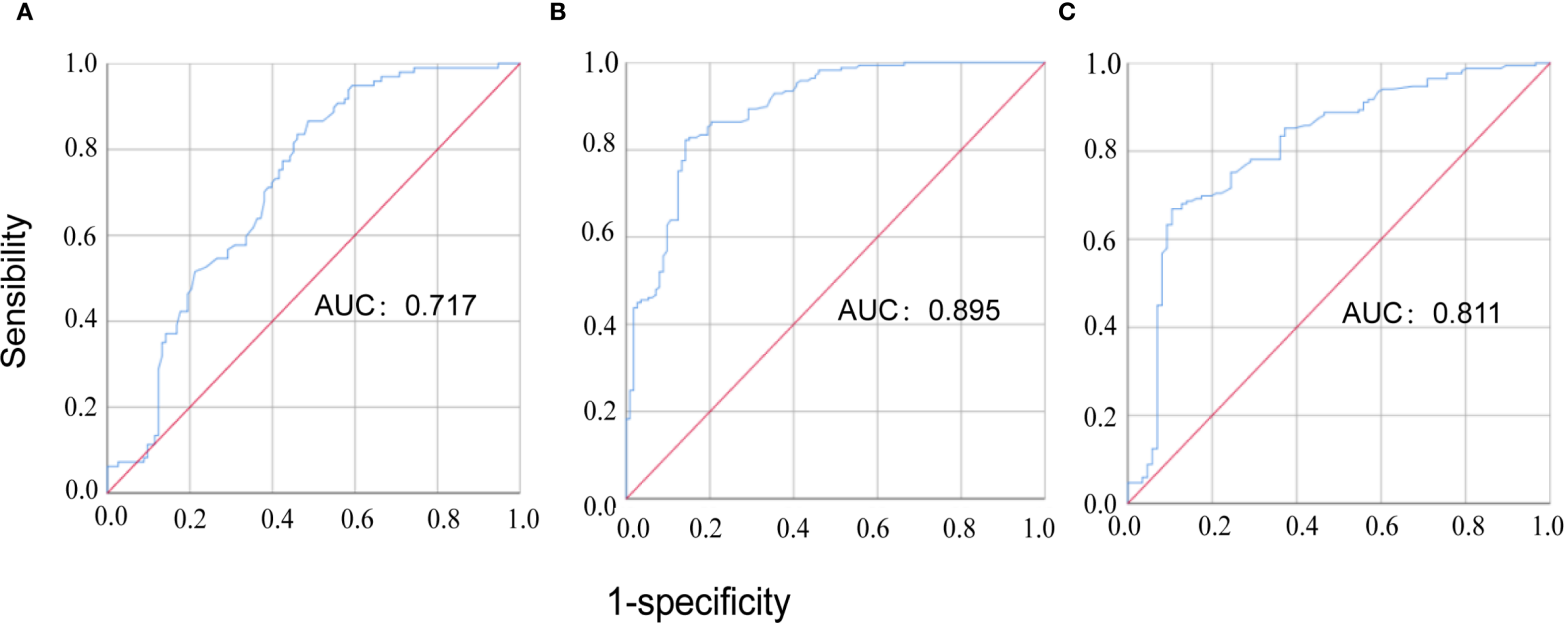
The ROC curve analysis of MFAP5 for discriminating CTD patients. **(A)** comparison between CTD-non-ILD patients and healthy controls; **(B)** comparison between CTD-ILD patients and healthy controls; **(C)** comparison between CTD-ILD patients and CTD-non-ILD patients.

The results of Spearman correlation analysis indicated that serum MFAP5 levels were significantly associated with disease activity in patients with CTD-ILD. Specifically, positive correlations were observed between MFAP5 levels and WBC (r = 0.274, P < 0.001), CRP (r = 0.718, *P*<0.001), RF (r = 0.224, *P*=0.012), C1q (r = 0.176, *P*=0.032), ESR (r = 0.169, *P*=0.030), IL6 (r = 0.357, *P*<0.001), and PCT (r = 0.381, *P <*0.001). Conversely, a negative correlation was found with ALB (r = -0.223, *P*=0.004) (**Table 2**). ROC curve analysis further demonstrated the predictive value of MFAP5 for CTD-ILD. For discriminating CTD-non-ILD, the cutoff value was 1.525 pg/mL, with AUCof 0.717, sensitivity of 86.6%, and specificity of 51.3%. For discriminating CTD-ILD, the cutoff value was 2.095 pg/mL, with an AUC of 0.895, sensitivity of 82.2%, and specificity of 85.8%. Additionally, for distinguishing CTD-ILD from CTD-non-ILD, the cutoff value was 2.480 pg/mL, with an AUC of 0.811, sensitivity of 66.9%, and specificity of 89.5% (**Figures 2A–C**). These findings underscore the high specificity of MFAP5 in discriminating CTD-ILD and CTD-non-ILD, highlighting it potential as a valuable biomarker in this context.

**Table 2:** Spearman correlations between serum levels of MFAP5 and biochemical indicators in CTD.

Characteristics	N	r	*p value*
Sex	169	0.11	0.167
Age	169	0.09	0.230
Duration of CTD	163	-0.02	0.772
RBC	169	-0.02	0.751
Hb	169	-0.08	0.318
WBC	169	0.274**	0.000
PLT	169	0.02	0.773
TBIL	167	0.00	0.981
DBIL	167	0.06	0.468
ALT	168	0.00	0.990
AST	168	-0.01	0.892
TP	168	-0.05	0.512
ALB	168	-0.223**	0.004
GLB	169	0.05	0.488
Glu	159	0.10	0.221
UREA	168	-0.10	0.217
CREA	168	-0.15	0.059
Egfr	166	0.09	0.228
TG	156	-0.07	0.378
CHOL	154	-0.14	0.082
ALP	167	0.11	0.159
GGT	166	0.274**	0.000
CK	168	-0.05	0.495
LDH	168	0.11	0.144
HBDH	167	0.11	0.155
D-dimer	138	0.09	0.288
CRP	168	0.718**	0.000
RF	126	0.224*	0.012
IgG	159	-0.01	0.885
IgA	160	0.09	0.257
IgM	160	0.11	0.153
C3	160	0.02	0.830
C4	159	-0.06	0.460
C1q	148	0.176*	0.032
ESR	166	0.169*	0.030
IL6	98	0.357**	0.000
PCT	83	0.381**	0.000

*P < 0.05, **P < 0.01.

### 3.3 MFAP5 is significantly upregulated in the lungs of CTD-ILD patients

**Figure 3:**
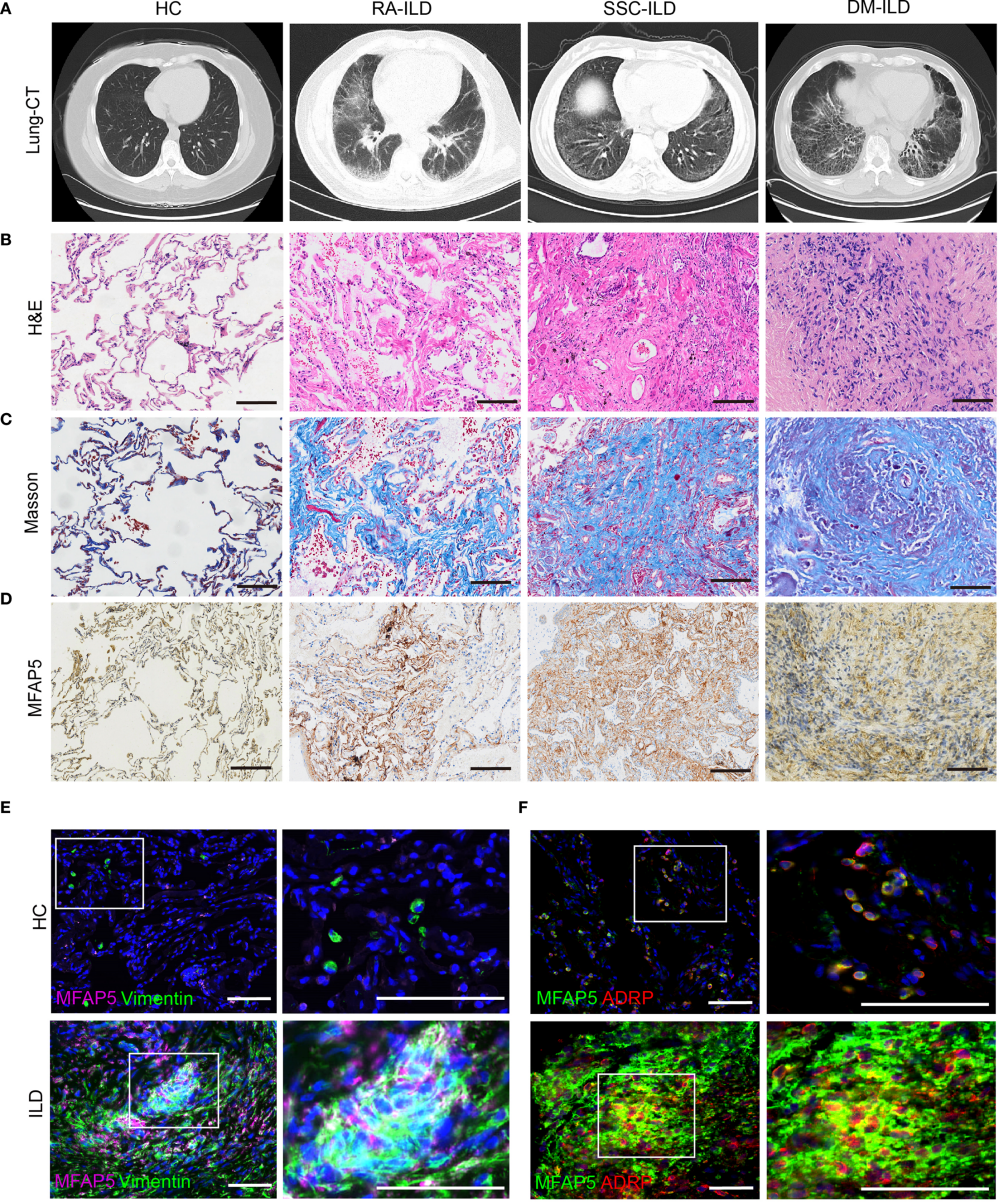
MFAP5 expression is elevated in the lungs of CTD-ILD patients. **(A)** ILD was confirmed via chest CT. **(B)** Lung biopsies from ILD patients underwent H&E staining, **(C)** Masson trichrome staining to assess collagen deposition, **(D)** and immunohistochemistry to quantify MFAP5 expression, scale bars, 100 μm. **(E)** Immunofluorescence staining of lung tissues revealed co-expression of MFAP5 with fibroblasts: green (Vimentin), pink (MFAP5), blue (DAPI). **(F)** Co-localization with adipose fibroblast subsets was observed: green (MFAP5), red (ADRP), blue (DAPI); scale bars, 50 μm.

Chest CT scans revealed symmetrical reticular or honeycomb changes in the posterior basal segments of both lower lobes in RA, SSC, and DM-ILD patients, with intralobular interstitial thickening, interlobular septal thickening (“grid-like” patterns), focal/diffuse ground-glass opacities, and fibrotic bands (**Figure 3A**). Histological analysis by hematoxylin-eosin (H&E) staining showed disrupted alveolar architecture, diffuse alveolar septal thickening, chronic inflammatory cell infiltration (lymphocytes, plasma cells), thickened small vessel walls with luminal stenosis, and perivascular fibrous encapsulation (**Figure 3B**). Masson trichrome staining demonstrated collagen fibers deposited in bundles, sheets, or reticular patterns within alveolar septa and bronchovascular bundles, with red fibrinoid exudates in alveolar spaces. Organizing lesions were observed in DM-ILD lungs, with blue-green collagen encasing small vessels and their surroundings (**Figure 3C**). MFAP5 protein expression was significantly higher in all three ILD subtypes compared to healthy controls, with the lowest levels in RA-ILD, moderate elevation in SSC-ILD, and maximal expression in DM-ILD, correlating with disease severity (**Figure 3D**). Immunofluorescence co-localization studies in ILD tissues showed MFAP5 expression in fibroblasts (**Figure 3E**), co-localization with adipose fibroblast (ADRP) subsets (**Figure 3F**), suggesting functional interplay between these cell types.

### 3.4 MFAP5 is upregulated in the BLM-induced mouse model of ILD

**Figure 4:**
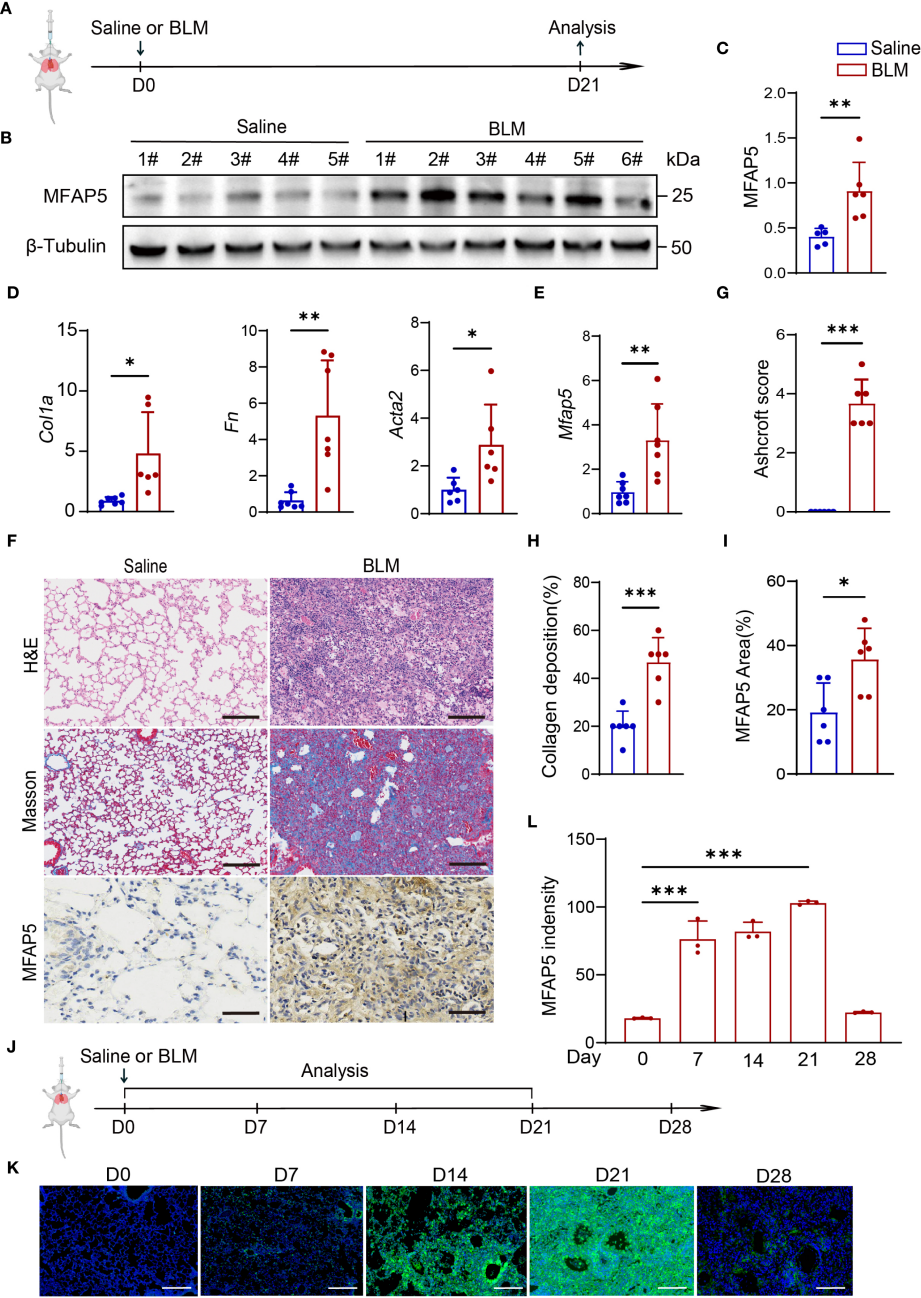
MFAP5 is upregulated in BLM-induced ILD model. **(A)** Schematic diagram of BLM-induced ILD. **(B)** Western blot analysis of MFAP5 protein expression in normal and BLM-ILD mouse lungs (n=5-6 independent experiments). **(C)** Quantification of band intensities. **(D)** qPCR analysis of *Fn*, *Col1a*, and *Acta2* mRNA levels in BLM-ILD and control lungs (n=6-7 independent experiments). **(E)** *Mfap5* mRNA expression in BLM-ILD vs. control groups. **(F)** H&E, Masson trichrome, and MFAP5 immunohistochemistry staining of lung sections from BLM-ILD and control mice. **(G)** Ashcroft scoring for ILD severity assessment. **(H)** Quantification of collagen deposition area. **(I)** Quantification of MFAP5 immunoreactivity. **(J)** Experimental timeline for lung tissue collection at different time points post-BLM instillation. **(K)** Immunofluorescence analysis of dynamic MFAP5 expression in BLM-ILD lungs: green (MFAP5), blue (DAPI). **(L)** Quantification of MFAP5 fluorescence intensity (n=3 independent experiments), (scale bars: 100 μm). Data are presented as mean ± standard error (n = 6 independent experiments). *p < 0.05; **p < 0.01; ***p < 0.001.

To validate MFAP5 expression *in vivo* and its role in ILD, the BLM-induced ILD mouse model was established via intratracheal instillation (**Figure 4A**). Protein and mRNA levels were analyzed in lung tissues from BLM-treated and saline control mice. Results showed MFAP5 protein levels were significantly higher in lung tissues of BLM-treated mice compared to saline controls (**Figures 4B, C**), indicating upregulation during BLM-induced ILD. mRNA analysis revealed increased expression of extracellular matrix (ECM) genes Collagen type Ia (Col1a), Fibronectin (Fn), and α-smooth muscle actin (α-SMA) in BLM-treated lungs (**Figure 4D**), The successful induction of the model was confirmed, as evidenced by the significantly higher MFAP5 mRNA levels in the bleomycin BLM-treated mice compared to those in the saline group (**Figure 4E**), corroborating the association between MFAP5 and ILD markers. Histological analysis showed alveolar damage, inflammatory cell infiltration, and thickened alveolar septa in BLM-treated lungs via H&E staining, accompanied by elevated Ashcroft scores (**Figures 4F, G**). Masson trichrome staining confirmed extensive collagen deposition in BLM-treated tissues, consistent with H&E findings (**Figures 4F, H**). Immunohistochemistry revealed that MFAP5 was primarily localized to the extracellular matrix, with significantly higher expression in BLM-treated lungs compared to saline controls (**Figures 4F, I**), further supporting its role in ILD. To investigate the dynamic changes of MFAP5 during fibrogenesis, lung tissues were collected at multiple time points from BLM-induced ILD mice (**Figure 4J**) and analyzed via immunofluorescence. Results showed that MFAP5 expression began increasing by day 7, peaked at day 21, and declined by day 28 post-BLM instillation (**Figures 4K, L**), indicating early activation and maximal expression during peak fibrosis. These findings demonstrate that MFAP5 is robustly upregulated in BLM-induced ILD, with expression levels tightly correlated with histological severity. Collectively, these data demonstrate a significant association between MFAP5 and ILD progression, suggesting its potential role and providing a foundation for future mechanistic studies.

## 4 Discussion

ILD are characterized by the pathological features of inflammation and fibrosis (45) and are associated with a poor prognosis, demonstrating a 5-year survival rate of less than 50% (17). In this study, we investigated molecular differences between fibroblasts derived from SSc-ILD and normal human lung fibroblasts. MFAP5 was identified as the most significantly upregulated gene. Elevated MFAP5 expression was observed in peripheral blood, lung tissue, and BALF from patients with CTD-ILD. Furthermore, the upregulation of MFAP5 in BLM-induced ILD models provides additional evidence supporting its involvement in ILD.

Fibroblast proliferation and activation constitute a pivotal pathogenic mechanism in ILD (46). Single-cell RNA sequencing revealed a distinct MFAP5hi fibroblast subset transcriptionally analogous to myofibroblasts (47). Our research findings also indicate that MFAP5 is co-expressed with fibroblasts, which suggests that MFAP5 may be involved in the process of fibroblast-to-myofibroblast transition. Additionally, other studies have shown that MFAP5 partially colocalizes with α-SMA, a marker of alveolar myofibroblasts, and enhances the activation of myofibroblasts (48). These activated myofibroblasts exhibit heightened proliferative capacity and excessive production of extracellular matrix components, including type I/III collagen and fibronectin (49, 50), driving pathological matrix deposition, parenchymal destruction, and loss of lung compliance. Besides, MFAP5 is also detected highly expressed in adipose tissue, and it mediates obesity-related adipose tissue remodeling and inflammation, promoting the fibrosis of adipose tissue (51). Subsequent studies in our research revealed the co-localization of MFAP5 with ADRP, suggesting a potential functional synergy between these proteins.

Moreover, MFAP5 is crucial for cell proliferation and differentiation (52, 53), and it promotes the proliferation and epithelial–mesenchymal transition (EMT) of cancer cells (54, 55). Additionally, MFAP5 interacts with various immune cell populations to regulate their activation and function (56, 57). Studies have shown that knockdown of MFAP5 significantly reduces the expression levels of inflammatory factors TGFβ1, IL1β, and IL-6, and our findings further demonstrate a correlation between MFAP5 and inflammatory markers (51).

Our study demonstrates a similar association between MFAP5 and ILD, revealing positive correlations between MFAP5 levels and WBC, LDH, C1q, CRP, ESR, and PCT, along with a negative correlation with anti-inflammatory immunoglobulin IgG. These findings are highly significant, as they link MFAP5 to key inflammatory indicators in ILD. Elevated WBC counts often indicate an inflammatory response in the body, while CRP and ESR, as classic inflammatory markers, show level changes closely related to the degree of inflammation. PCT and LDH levels significantly increase under inflammatory conditions such as bacterial infections. The positive correlation of MFAP5 with these indicators suggests its potential involvement in the inflammatory processes of ILD patients. Inflammation is a key driver in the development and progression of fILD, as persistent inflammatory stimuli can activate lung fibroblasts and promote collagen deposition. Therefore, MFAP5 may not only reflect the degree of inflammatory activity in ILD but could also be potentially linked to the initiation or progression of fILD. Furthermore, studies have found that MFAP5 expression increases with age in ILD patients. Aging itself is a risk factor for ILD with a higher incidence and potentially more rapid progression among older adults (49). As age increases, the body’s immune balance is easily disrupted (58), and a chronic low-grade inflammatory state becomes more common. This may not only influence MFAP5 expression but elevated MFAP5 levels could further drive the progression of ILD by exacerbating inflammation and promoting fibroblast activation.

Combined with the positive correlation between MFAP5 levels and the extent of ILD involvement, as well as ROC analysis confirming its predictive ability for CTD-ILD, these findings indicate that MFAP5 may reflect the progression of ILD, offering value in both disease prediction and condition assessment. Given its associations with inflammatory and complement markers, it is speculated that MFAP5 plays a role by participating in the inflammation-fibrosis process. In the BLM-induced mouse model of ILD, the expression of MFAP5 in lung tissue showed a positive correlation with ILD markers such as Col1a, Fn, and α-SMA, and it further confirmed that MFAP5 is involved in the pathogenesis of ILD. These consistent observations across clinical and experimental systems implicate MFAP5 as an active participant in ILD pathogenesis.

In the present study, a multi-dimensional approach was adopted to detect the expression level of MFAP5 in patients with CTD-ILD. Comparative analysis with patients’ clinical indicators demonstrated that MFAP5 could serve as a biomarker for diagnosing and monitoring the progression of ILD. Nevertheless, this study has certain limitations. First, we did not validate the performance of this biomarker in BALF samples obtained from CTD-ILD patients without concurrent infection. The specificity of MFAP5 in CTD-ILD patients with concurrent infection still requires confirmation through further prospective studies, which represents a critical issue to be resolved before this biomarker can be translated into clinical practice. Additionally, to strictly control the interference of smoking, an important confounding factor, on the pulmonary microenvironment and biomarker expression, our study cohort was restricted to non-smokers. Consequently, whether the expression pattern of MFAP5 in smoking CTD-ILD patients is consistent with the findings of this study warrants further investigation.

## Data Availability

The datasets presented in this study can be found in online repositories. The names of the repository/repositories and accession number(s) can be found in the article/supplementary material.
